# A Review: The Beauty of Serendipity Between Integrated Circuit Security and Artificial Intelligence

**DOI:** 10.3390/s25154880

**Published:** 2025-08-07

**Authors:** Chen Dong, Decheng Qiu, Bolun Li, Yang Yang, Chenxi Lyu, Dong Cheng, Hao Zhang, Zhenyi Chen

**Affiliations:** 1 College of Computer and Data Science, Fuzhou University, Xueyuan Rd., Fuzhou 350108, China; dongchen@fzu.edu.cn (C.D.); 231020039@fzu.edu.cn (D.Q.); 231020035@fzu.edu.cn (B.L.); 241020013@fzu.edu.cn (C.L.); chengdong2021@foxmail.com (D.C.); 2 Fujian Provincial Key Laboratory of Network Computing and Intelligent Information Processing, Fuzhou University, Xueyuan Rd., Fuzhou 350116, China; 3 School of Computing and Information Systems, Singapore Management University, Singapore 178902, Singapore; yang.yang.research@gmail.com; 4 Computer Science and Engineering, University of South Florida, Tampa, FL 33620, USA; zhenyichen@usf.edu

**Keywords:** hardware security, integrated circuits security, artificial intelligence, integrated circuits, hardware Trojans

## Abstract

Integrated circuits are the core of a cyber-physical system, where tens of billions of components are integrated into a tiny silicon chip to conduct complex functions. To maximize utilities, the design and manufacturing life cycle of integrated circuits rely on numerous untrustworthy third parties, forming a global supply chain model. At the same time, this model produces unpredictable and catastrophic issues, threatening the security of individuals and countries. As for guaranteeing the security of ultra-highly integrated chips, detecting slight abnormalities caused by malicious behavior in the current and voltage is challenging, as is achieving computability within a reasonable time and obtaining a golden reference chip; however, artificial intelligence can make everything possible. For the first time, this paper presents a systematic review of artificial-intelligence-based integrated circuit security approaches, focusing on the latest attack and defense strategies. First, the security threats of integrated circuits are analyzed. For one of several key threats to integrated circuits, hardware Trojans, existing attack models are divided into several categories and discussed in detail. Then, for summarizing and comparing the numerous existing artificial-intelligence-based defense strategies, traditional and advanced artificial-intelligence-based approaches are listed. Finally, open issues on artificial-intelligence-based integrated circuit security are discussed from three perspectives: in-depth exploration of hardware Trojans, combination of artificial intelligence, and security strategies involving the entire life cycle. Based on the rapid development of artificial intelligence and the initial successful combination with integrated circuit security, this paper offers a glimpse into their intriguing intersection, aiming to draw greater attention to these issues.

## 1. Introduction

Integrated circuits (ICs) are the core foundation of cyber-physical systems (CPSs). The booming development of ICs has brought endless possibilities to human society, such as supercomputers, intelligent robots, autonomous vehicles, metaverse devices, or even brain–computer interfaces. On a small silicon chip, tens of billions of components are integrated, enabling quite complex functions, including System on a Chip (SoC) and Network on a Chip (NoC).

In order to achieve lower costs, shorter time to market, and a better combination of high-tech innovations from various countries, the design and manufacturing life cycle of ICs is based on a global supply chain model. Stated differently, numerous untrusted third parties are involved in design and manufacturing processes, such as suppliers of intellectual property (IP) cores and electronic design automation (EDA) tools, foundries, and also internal employees. Such a model gives rise to various IC security issues. For instance, it creates opportunities for adversaries to stealthily insert malicious circuits, known as hardware Trojans (HTs), into the original circuit.

Currently, catastrophic security issues caused by ICs occur frequently. “IC security” is no longer an unfamiliar concept, as it relates to personal property and privacy, and even a nation’s economy and politics. Determining how to create secure and reliable ICs is an unavoidable research topic for humanity. Indeed, the early academic community has conducted research on IC security, such as channel analysis, reverse engineering, and logic analysis. The main idea of these methods is to carry out comparisons with a golden reference (also called a golden chip) to determine whether the chip under testing has any abnormalities.

But, for today’s ultra-highly integrated chips, novel ideas must be pursued for the following three reasons. First, it is very difficult to measure the tiny abnormalities in current and voltage caused by very small malicious circuits on a chip composed of tens of billions of components. Second, from the perspective of computability, traditional methods are inefficient or even unfeasible. Finally, it seems almost impossible to obtain a golden reference chip for a device composed of tens of billions of components, especially with the participation of multiple untrusted third parties.

Artificial intelligence (AI), especially machine learning (ML), enables computers to learn from large amounts of past known data and make predictions or decisions for unknown tasks. Clearly, AI can perfectly address the above research needs of today’s IC security, namely, eliminating the need for a golden chip, achieving high accuracy, and handling intensive computing.

Gratifyingly, in 2008, the first AI-based IC security paper emerged [1]. Many studies have focused on combinations of AI and IC security, including c (RF), XGBoost, support vector machine (SVM), and even some deep learning approaches, such as convolutional neural network (CNN), textCNN, graph neural network (GNN), etc. However, although these studies are only beginning to attract widespread attention, there is still no comprehensive and systematic survey of them.

To have a more comprehensive understanding of the security issues faced by ICs and summarize the latest research progress about IC security with AI in recent years, this paper comprehensively reviews the studies and achievements in IC security and related defense approaches and discusses open issues. The main contributions of this paper are as follows:

- Reviewing the design and manufacturing process of ICs and discussing the security risks encountered in these processes.
- Summarizing the attack characteristics and destructiveness of HTs and introducing the design principles and details of information leakage, denial of service, degraded performance, and function change HTs.
- Comparing the AI-based HT detection technologies, describing the details of different approaches during detection, listing the features used in the detection process.
- Illustrating the challenges and difficulties faced by current AI-based technologies in the field of ICs security and outlining the open issues that need to be addressed in future research.

The rest of the paper is organized as follows. Section 2 describes the background knowledge of ICs, including their evolution, integration scale, and the design and manufacturing processes. Section 3 discusses the security risks of ICs, mainly introducing the threat sources and classifications of HTs. Section 4 introduces the definition and working principles of HT, and also HT on special chips. Section 5 provides a detailed introduction and discussion on the attack models associated with the impact of HTs. Section 6 and Section 7 focus on organizing and comparing HT defense technologies based on traditional and advanced ML methods, respectively. Challenges and future work are presented in Section 8. The conclusion is summarized in Section 9.

## 2. Background

This section presents the background knowledge of IC, including its evolution, integration, design and manufacturing.

### 2.1. Preliminary Concepts

To facilitate comprehension of the terminology used throughout this review, Table 1 provides a summary of the commonly used terms and their corresponding abbreviations. Familiarity with these terms will be essential for understanding the technical discussions and methodologies presented in the following sections.

### 2.2. Origin and Evolution

In 1947, Bardeen, Shockley, and Brattain invented the first point-contact transistor at Bell Labs [2]. This significant advancement laid a solid foundation for the emergence of ICs.

In 1958, Jack Kilby of Texas Instruments proposed the concept of ICs [3], and utilized silicon blocks with oxide layers to fabricate capacitors, employed diffusion techniques to create resistors, and integrated silicon transistors to construct phase-shift oscillator circuits. These pioneering efforts led to Kilby being awarded the Nobel Prize in Physics in 2000.

In 1959, Noyce of Fairchild Semiconductor Company in the United States successfully developed silicon ICs using innovative planar technology [4], realizing monolithic ICs.

In 1960, with the breakthrough of MOSFET materials and gate stability technology, the first MOS IC was born. Two years later, the world’s first commercial IC was developed.

In 1965, Moore, the co-founder of Intel Corporation, discovered a pattern while compiling observations on computer memory and proposed the famous Moore’s Law [5].

In 1966, the emergence of CMOS ICs and the first gate array promoted the development of large-scale ICs. Subsequently, in 1971, Intel launched the first microprocessor, ushering in a new era of computers.

Nowadays, an IC is a microelectronic circuit that connects multiple electronic components (such as transistors, resistors, capacitors, etc.) and integrates them into a small piece of semiconductor material [6]. A tiny IC can contain an entire computing system, or even a network, capable of performing intricate and complex functions. Incredibly, from its origins to its current state, the IC’s evolution has spanned less than a hundred years, which can be called a true miracle of human scientific and technological advancement.

### 2.3. Circuit Scale and Integration

Depending on different purposes and manufacturing processes, a single IC may contain thousands or even billions of transistors, resistors, capacitors, and diodes. ICs can be divided into different scales according to the scale of integration of electronic components [7].

As shown in Table 2, they are called Small-Scale Integrated Circuits (SSIs), Medium-Scale Integrated Circuits (MSIs), Large-Scale Integrated Circuits (LSIs), Very-Large-Scale Integrated Circuits (VLSIs), Ultra-Large-Scale Integrated Circuits (ULSIs), and Giga-Scale Integrated Circuits (GSIs).

As of 2024, the most integrated ICs are typically the latest high-end processors or SoCs, such as NVIDIA H100 Tensor Core GPU, containing 80 billion transistors [8], Apple M2 Ultra, containing more than 134 billion transistors [9], and Intel Xeon Max Series, containing about 100 billion transistors [10].

### 2.4. Design and Manufacturing Flow

**Figure 1:**
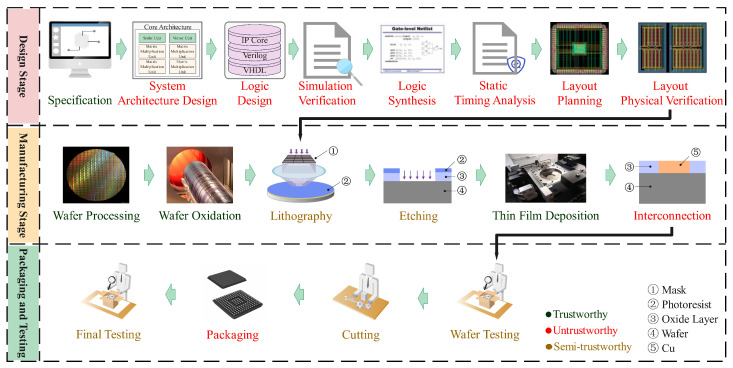
Overall process of IC design and manufacturing.

As shown in Figure 1, the ICs’ whole life circle can be approximately divided into three major stages: design stage, manufacturing stage, and packaging and testing stage [11].

#### 2.4.1. Design Stage

The design stage involves using a systematic and standardized process to design a circuit with complete functionality and excellent performance. The final output is a GDSII format layout file (Graphic Data System II, GDSII). The design process can be roughly divided into seven steps: specification, system architecture design, logic design, simulation verification, logic synthesis, static timing analysis, layout planning, and layout physical verification [12,13,14].

#### 2.4.2. Manufacturing Stage

The manufacturing stage aims to convert the layout design file into a physical IC, which involves a large number of processes and procedures, such as lithography and etching, covering disciplines in the fields of physics, chemistry, and microelectronics [15]. The complex and diverse production process can be roughly divided into six steps, including wafer processing, wafer oxidation, lithography, etching, thin film deposition, and interconnection.

#### 2.4.3. Packaging and Testing Stage

The packaging and testing stage is responsible for testing the circuits on the wafer to ensure they meet the design specifications. The ICs that pass the test are sent to the market after assembly and finally deployed in electronic systems. The packaging and testing stage includes four steps: wafer testing, which checks functionality and parameters; cutting, which separates the wafer into individual circuits; packaging, which protects the circuits and provides electrical connections; and final testing, which verifies overall functionality and performance [16,17].

## 3. Integrated Circuit Security Risks

This section focuses on security threats to IC; then, the classification of security threats is presented.

### 3.1. Source of Security Threats

The complexity of IC design, manufacturing, and distribution has led the entire electronics industry to shift to a global business model in which third-party resources in hardware circuit design, such as third-party IPs and EDA tools, have been widely used in modern circuit design and manufacturing, and have become an indispensable part of the contemporary circuit design and manufacturing process [18,19,20].

With the advancement of process technology and the global development of the electronics industry, IC security is confronting significant challenges, and CPS security has become an increasingly important issue. The following are possible sources of security threats:

#### 3.1.1. Untrustworthy Third-Party IP Suppliers

IP cores have greatly reduced manufacturing costs, improved the success rate of chip design, and reduced time-to-market (TTM). It is impossible for IC design and manufacturing companies to develop all the necessary IP cores independently, so they need to purchase some third-party IP cores [21], and these IP cores may contain malicious circuits [22].

#### 3.1.2. Untrustworthy Design Companies

Global supply chains often involve multiple countries and regions, as well as numerous participants and links [23]. Assume that ICs are designed by untrustworthy companies and manufactured by trusted foundries, which will also make the entire IC supply chain untrustworthy.

#### 3.1.3. Untrustworthy Original Equipment Manufacturers

Fabless design companies do not own their wafer manufacturing plants; they outsource production to third-party original equipment manufacturers (OEMs) [24]. Untrustworthy OEMs may perform attacks during the lithography, etching, and interconnection stages. The attacker manipulates the lithography mask or implants HTs in the wire routing.

#### 3.1.4. Untrustworthy Material Suppliers

The materials that the foundry needs generally include silicon wafers, photoresists, substrate materials, special gases, and other raw materials. Untrustworthy material suppliers can cooperate with the foundry to implement HT attacks or bypass the foundry to implement HT attacks [21,25].

#### 3.1.5. Untrustworthy EDA Tool Suppliers

EDA software is an important tool used in the design phase of ICs [19], helping engineers design, analyze, and optimize electronic systems. The EDA tools provided by untrustworthy suppliers are a black box to designers, which may be subject to security threats, such as HTs.

In addition, the risk source of security issues may also be a combination of the above-mentioned risk sources, and the internal employees are also a source of security hazard.

### 3.2. Attack Approaches for ICs

In the early days, the attacks aimed at hardware systems mainly involved mathematical analysis and the cracking of related encryption algorithms, essentially, which did not directly attack the hardware itself. Nowadays, as human life is deeply involved in CPS, numerous novel physical attacks have emerged, especially with the rise of ML. The most common attack approaches include side-channel attacks, fault injection attacks, reverse engineering, and HTs, with HT being one of the primary methods.

#### 3.2.1. Side-Channel Attack

Side-channel attacks use the bypass information of encrypted devices to crack the hardware system’s security during the encrypted device’s operation [26]. The current hardware device execution tasks, such as instruction operation time and instantaneous power consumption of the system, generally generate the bypass information. Because the side-channel attack is non-invasive, passive, and can usually be executed with relatively cheap devices, it seriously threatens the security of most encrypted hardware devices. The scope of such devices, ranging from personal computers to small embedded devices like smart cards and radio frequency identification devices (RFIDs), according to leaked information, includes common bypass information attacks, such as power consumption attacks, electromagnetic attacks, and time attacks.

#### 3.2.2. Fault Injection Attack

Fault injection technology is a method of evaluating and testing system reliability [27]. It involves inserting faults in the system and monitoring the system to determine its response to faults. It can be divided into hardware-based fault injection, software failure injection, simulation-based fault injection, and mixed fault injection. However, fault injection attacks are a technology in which injection failure through physical methods destroys hardware system behavior. In 1997, Boenh et al. [28] studied and proposed a fault injection attack model using random hardware failure to solve various password schemes. The proposed model can almost destroy the implementation of all password algorithms on the hardware and the security of the password equipment, which has a huge impact on the security field of password equipment. Failure injection attacks are not limited to attacks on password hardware but can also be used to attack any IC.

#### 3.2.3. Reverse Engineering

RE technology is mainly aimed at post-silicon chips. It is the technology with the best detection rate and the most accurate among all the HT detection technologies proposed so far. It can be divided into five parts [29]: disassembling the chip package, dissolving the metal layer, scanning the exposed metal layer with a microscope or other technology and saving the image, marking the main connectors on the image, and, finally, restoring the circuit structure description at the netlist level based on the marked image. There are two ways to choose RE technology, which are to directly compare pixels on the image extracted by RE [30] or to divide the image into a netlist form, extract features from the netlist, and use the ML model to classify and judge HT [31].

#### 3.2.4. Hardware Trojan Attack

The standard definition of the HT was proposed by the IBM Research Center in 2007 [32,33]. The HT refers to the malicious circuit or harmful alterations to the original circuit from the chip design stage to the chip packaging test stage. Differing from software viruses and software Trojans, the HT cannot be easily eliminated through the firmware. Therefore, HT is more harmful to the computer system. Attackers often use methods to add unnecessary functions to the IC design into HT. The design of the HT does not have a standard process, and the method adopted depends on the target and available resources of the attacker. Nevertheless, Ref. [34] still classifies HTs based on an abstract level, activation mechanism, physical attributes, insertion, position, attack mode, physical attributes, logical structures, and insertion fields.

#### 3.2.5. Other Attacks

In addition to the above attacks, the security of the hardware system is also threatened by various vulnerabilities, including unknown software and hardware vulnerabilities, preset software and hardware back doors, and virus attacks [35]. IEEE SPECTRUM reported on the 2007 missile attack. The defensive radar system did not make necessary early warning. Scientists analyzed that the “Trojan” or “back door” was implanted in the commercial chip used by the radar system during the manufacturing process. The nuclear power plant attack in 2012 by the Stuxnet virus, triggered by a backdoor circuit, occurred while the information system was physically isolated. Backdoor attacks are a type of security attack in which attackers implant a backdoor in a system, software, or hardware to gain unauthorized access to or control over the target system [36]. A backdoor is a hidden entrance or channel that allows an attacker to bypass normal authentication and authorization mechanisms and secretly access or manipulate the system.

## 4. Learn About Hardware Trojans

Among all the risks, HTs are one of the primary security issues. As the design, manufacturing, packaging, and testing processes of ICs are very complex and involve multiple steps and stages, attackers have chance to implant HTs at different stages. Moreover, the use of IP cores and EDA tools has increased the risk of HTs implanted in ICs [37]. As expected, when the triggering conditions are met, HTs may lead to catastrophic consequences for different purposes. To resist the impact of HTs, understanding HTs is essential.

### 4.1. Definition of Hardware Trojan

Generally, HT refers to malicious circuits or harmful alterations to the original circuit that exists from the IC design stage to the packaging test stage [33], which may cause functional changes, information leakage, denial of service, or even irreversible damage [38]. HTs lurk in the dark, causing system failures under certain conditions or leaking confidential information through bypasses, or causing other consequences due to their small circuit size, rare activation, strong concealment, and significant destructive potential [34,39,40].

It is important to note that maliciousness is a typical characteristic of HT, which is a different characteristic from the security rather than reliability issue.

### 4.2. Working Principle and Structure

**Figure 2:**
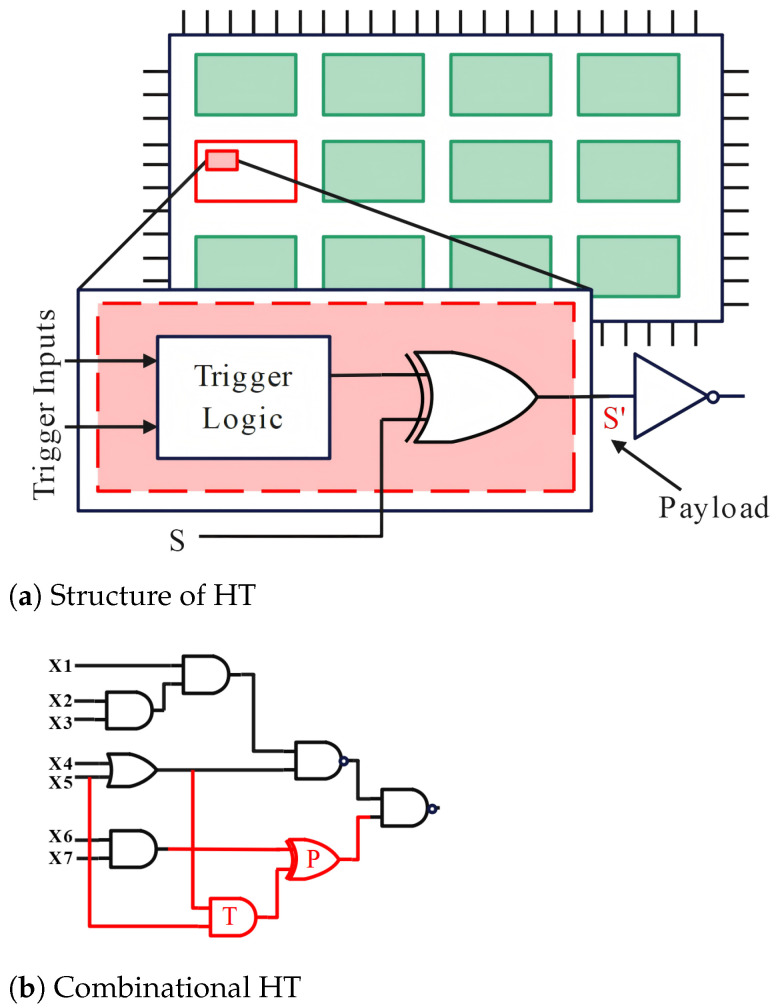

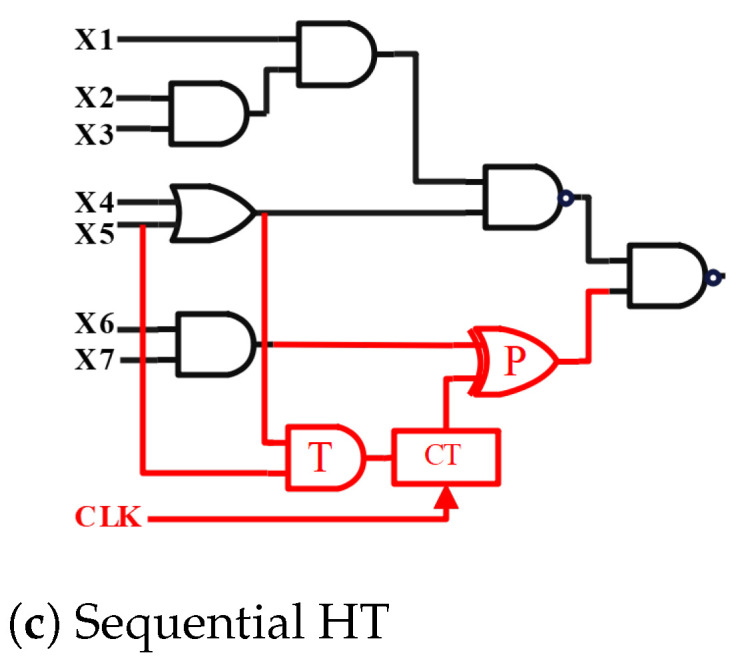
Structure and classification of HT. The red regions represent hardware Trojans.

HTs mainly comprise two functional components: trigger logic and payload [41], as shown in Figure 2a. The trigger logic activates the payload by monitoring input signals, data/control buses, register status, or set working time. The payload logic is the attack unit of the HT, responsible for executing the attack behavior.

#### 4.2.1. Combinational and Sequential HTs

Banga et al. [42] classified HTs into combinational and sequential Trojans based on circuit type, as shown in Figure 2b,c.

Bhunia et al. [43] explained combinatorial and sequential Trojans in more detail. Combinatorial Trojans do not contain any state elements (such as triggers or latches) and only depend on the simultaneous occurrence and fault triggering (by flipping the signal ER) of a set of rare node conditions (such as predefined values on a set of nodes). Sequential Trojans are triggered by a K-bit counter. The Trojan circuit implements the attack by monitoring the clock signal CLK. When the count value reaches $2^{K}-1$, the HT is triggered, and the value of the modified node ER is changed to ER*.

#### 4.2.2. Digital and Analog HTs

According to the different types of signals in the ICs, HT can be further divided into digital and analog Trojans. Digital Trojans include combinatorial and sequential types, while analog Trojans are triggered by sensors sensing changes in external conditions or voltage changes.

### 4.3. Hardware Trojans on Special Chips

**Figure 3:**
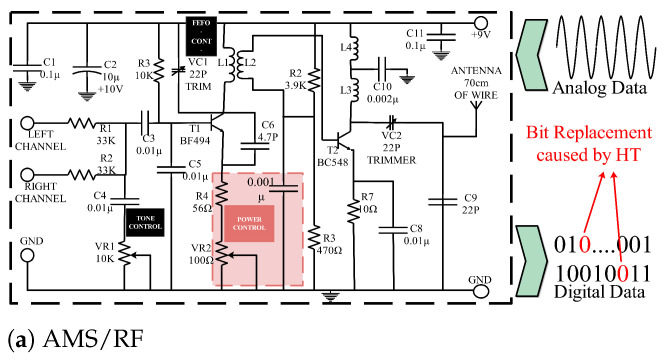

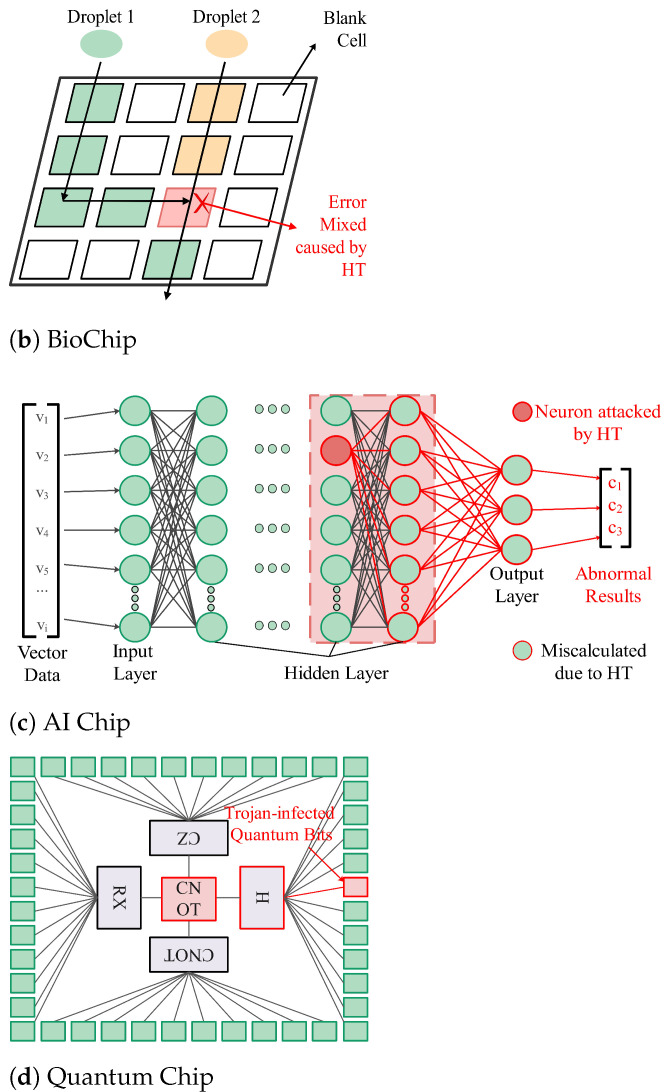
HT on special chips. The red regions represent hardware Trojans.

IC manufacturing technology has developed rapidly, and the style of chips is no longer limited to traditional circuits. New chips such as analog/mixed-signal (AMS) and radio frequency IC (RF IC), biochips, and artificial intelligence chips (AI chips) have emerged one after another [44,45,46]. However, new chips have not escaped the attack of HTs. As illustrated in Figure 3, HT can manifest in diverse forms and infiltrate different chip types. In [34], the paper discussed the impact of HTs on AMS/RF, biochips, and AI chips.

#### 4.3.1. AMS/RF

An AMS circuit consists of two parts: analog and digital. The combination of triggers and payloads of HTs in AMS circuits can be more diverse, including analog triggers plus digital payloads or analog triggers plus analog payloads. RF ICs belong to AMS circuits and are widely used in many wireless network environments. Attackers implant Trojans in the forward error correction (FEC) encoder in the wireless router of the transmitter so that the correct bits are replaced during the information transmission process, resulting in the receiver receiving incorrect information.

#### 4.3.2. Biochip

A biochip is a microfluidic device called a lab-on-a-chip (LOC), and biologists perform a series of biochemical reactions with reagents on the biochip [47]. During the chip production process, attackers can create malicious biochips by implanting HTs, which may cause changes in the flow path of droplets. Incorrect or accidental mixing of droplets can easily destroy biological experiments and waste reagents. If an HT is implanted in it, it will cause great trouble to the biochip’s security monitoring and normal operation. In addition, some HTs may damage the valves in the biochip, accelerate their aging, or cause switching errors and reagent contamination.

#### 4.3.3. AI Chip

AI chips can be divided into two categories: traditional AI chips and new AI chips. As traditional AI chips still use CMOS components, they face the same risks as ICs [48]. New AI chips generally use new devices such as non-volatile memory, while, they are also face the risk of HTs. Attackers can inject a small HT circuit into the neural network to change the output parameters of the hidden layer, resulting in subsequent calculation errors. For example, in the paper [49], the authors designed two HTs and implanted them into a neuromorphic system based on RRAM (resistive random access memory). These Trojans are designed to dynamically change the activation threshold of neurons, thereby causing changes in the function of the neural network.

#### 4.3.4. Quantum Chip

In addition, with the emergence of quantum computers [50], many current cryptographic algorithms have become vulnerable. Researchers at the University of Munich in Germany have, for the first time, created a computer chip that can apply post-quantum cryptography. The chip can efficiently implement post-quantum cryptography, thereby resisting attacks launched by future attackers using quantum computers. Some researchers believe that for post-quantum cryptography, the threat posed by so-called HTs still exists. If an attacker implants a Trojan circuit into the chip design during the chip design or manufacturing stage, serious consequences will occur [51,52].

## 5. Hardware Trojan Attack Model

Currently, serious consequences caused by HTs include, but are not limited to, leak information, denial of service, degraded performance, and change functionality.

HT benchmarks are usually used as datasets for ML model training in AI-based methods of detecting HTs. This section introduces different types of HT starting with the HT benchmarks and summarizes several different purposes HTs on Trust-Hub [53]. Table 3 shows their respective characteristics.

### 5.1. Leak Information

With the purpose of information leakage using HTs, attackers usually insert covert channels in circuits or maliciously obtain high access rights to obtain secret information stored in storage areas with high access rights. The secret data involved in the theft usually includes encryption keys, user passwords, and other important confidential information.

There are many types of leak information HTs. Depending on different chip architectures, application scenarios, etc., HTs can be implanted in different functional modules of ICs. At the same time, they can also be implanted in different design and manufacturing stages. For example, Refs. [55,56,57] insert information leakage HTs into the pre-silicon design of ICs, and Refs. [58,59] implant HTs during the manufacturing process of ICs.

Currently, the information-leakage HTs commonly used for model training include AES-T100, PIC16F84-T300, S38584-T300, etc.

### 5.2. Denial of Service

Similar to leak information HTs, denial-of-service HTs can also be implanted at various stages of IC design or manufacturing. This type of HT can be activated under certain conditions, causing the system to refuse to perform certain services, including constantly rotating shift registers, reducing clock frequencies to slow down some circuits, fixing output signals, etc. Even in the on-chip network NoC, denial-of-service HTs can cause transmitted data packets to be retransmitted, causing resource congestion or deadlock, thereby achieving the purpose of denial-of-service.

Denial of service HTs can be implanted at different design and manufacturing stages. For example, in [60], an HT implanted at the manufacturing stage is proposed. This HT can change the doping polarity of the transistor during the doping stage, thereby accelerating the aging of the device during the operation of the IC and eventually causing part of the circuit to fail, thereby achieving the purpose of denial of service. Unlike [60,61], it implants an HT circuit at the design stage. This HT runs in the NoC and injects faults by identifying specific data packets, thereby achieving denial of service by creating deadlocks or consuming network resources.

Currently, the denial-of-service HTs commonly used for model training include AES-T1800, B15-T100, BASICRSA-T200, WB_CONMAX-T100, etc.

### 5.3. Degrade Performance

A performance-degrading HT is a malicious implanted hardware component designed to reduce the performance of the circuit or system when it does not cause immediate failure. The form includes widening or narrowing the critical path, issuing a flash sleep command through the memory controller to place the connected flash device into sleep mode, slowing down the expected path through the ring oscillation of the ring oscillator (RO), etc.

Degraded-performance HTs can be implanted at multiple stages. In the design phase, Ref. [62] proposed a new HT attack scheme in which the test infrastructure is the carrier for infecting analog IP cores. After activation, the Trojan transfers the load module to the target analog IP core through the reconfigurable scan network, bypassing other IPs. Its load module includes bits used to activate the DfT structure or analog IP core interface, and digital keywords that change the behavior of the analog IP core. The digital keywords may cause the performance of the IP core to degrade or malfunction. In addition, Trojans such as ETHERNETMAC10GE-T100 and MULTPYRAMID-T100 are implanted at the manufacturing stage.

Currently, common performance-degrading HTs include ETHERNETMAC10GE-T100, MEMCTRL-T100, MULTPYRAMID-T100, S35932-T300, and others.

### 5.4. Change Functionality

Function-changing HTs are a type of HT that achieves malicious purposes by changing the intended functions of ICs. This type of HT usually consists of a trigger module and a load module. To achieve better concealment, it will only be triggered under specific conditions. Function-changing HTs include attacking the encryption module to make it invalid, using an “OR” gate to change the original netlist design, and using a multiplexer in the load module to change the output of the original netlist. At the same time, denial-of-service HT attacks often lead to functional changes.

Function-changing HTs can be implanted at multiple stages. Ref. [63] proposes an HT independent of the IP core. As this type of HT does not rely on the original circuit design, it can be implanted at the manufacturing stage. At the same time, Function-changing HTs can also be implanted at the design stage. On Trust-Hub, Function-changing HTs are mainly implanted in the netlist file as gate-level netlists.

Common Function-changing HTs include AES-T2300, B19-T100, S15850-T100, and others. These Trojans are often used for model training.

## 6. Defense Strategies Based on Traditional Machine Learning

With the widespread application of AI in the IC field, various defense strategies for ICs that utilize ML methods have emerged. This work broadly categorizes into these strategies based on traditional ML and advanced ML. Traditional ML-based methods rely on manual feature engineering, extracting structural and electrical features from circuits and feeding them into shallow models that operate on vectorized data, which limits their ability to capture internal structural dependencies. In contrast, advanced ML-based defense strategies can automatically learn high-level feature representations from raw or minimally preprocessed circuit data and are capable of handling non-Euclidean data structures such as circuit netlists. Moreover, advanced methods demonstrate superior capabilities in modeling the topological structures and temporal dependencies within circuits, making them more expressive and effective in complex IC defense scenarios.

HT detection based on traditional ML includes SVM, RF, PCA, KNN, and K-means. Table 4 summarizes the HT defense strategies based on traditional ML.

### 6.1. SVM-Based

SVM is a commonly used binary classification algorithm in ML. When two data types are linearly separable, the SVM algorithm finds a suitable hyperplane to separate them and uses the hyperplane to classify new samples [86]. SVM-based methods are widely used in HT detection.

Hasegawa et al. [64] proposed five feature dimensions for pre-silicon training: logic gate input, trigger input and output, primary input and output. The authors used random initialization for the SVM kernel bandwidth γ and penalty coefficient *C*, applying random search followed by grid search to optimal parameters for maximizing TPR.

Vashistha et al. [65] applied SVM to physical inspection by extracting shape features from N-type and P-type regions of 28 nm ICs after image segmentation. Key features included centroid coordinates, distance between centroids, width, height, and distance between N-type and P-type regions. The radial basis function (RBF) was used as the SVM kernel, with γ = 1000 and *C* = 0.005, balancing complexity and smoothness to minimize classification errors.

In addition, Hu et al. [66] applied SVM to side-channel analysis using a Gaussian kernel, optimizing the parameters through cross-validation by initially testing with a broad range (0.001 to 500) and refining it based on training results. Kulkarni et al. [67] utilized SVM for runtime Trojan detection in multi-core platforms, monitoring changes in packet transfer paths (PTPs) between source (SC) and destination cores (DCs).

In the aforementioned methods, both Hasegawa et al. [64] and Vashistha et al. [65] focus on circuit features, with the former emphasizing detection in the pre-silicon stage and the latter focusing on detection in the post-silicon stage. In contrast, Hu et al. [66] and Kulkarni et al. [67] rely on side-channel analysis and runtime data, respectively, highlighting the need for detection in real-time systems and deployed hardware. In these methods, SVM serves as the classifier. However, SVM does not possess feature extraction capabilities, and its performance is highly dependent on the quality of the input features and the effectiveness of the preprocessing process. Therefore, while SVM plays an important role as a classification tool in these methods, its actual effectiveness largely depends on the quality of feature engineering and data preprocessing.

### 6.2. RF-Based

Before introducing the RF method, the decision tree is discussed first. Because of its simple structure, efficient algorithm, and fast construction, the decision tree is an ideal choice for building an ensemble learning model. Therefore, existing HT defense solutions based on decision trees are usually combined with ensemble learning.

Pan et al. [68] proposed a gate-level HT detection method combining Shapley value analysis with decision trees, utilizing 51 HT features from [70] and incorporating dynamic power changes and rare switch counts from simulations [87], resulting in 55 candidate features. The Shapley values are used to analyze and evaluate the contribution of each feature in each iteration. The final prediction is made through a voting process between decision trees, and the weights are adjusted based on the accuracy.

The RF algorithm is an ensemble learning algorithm that improves the accuracy and robustness of predictions by building multiple decision trees and voting or averaging them. This method is widely used in HT detection.

Hasegawa et al. [70] proposed an RF-based gate-level network list HT detection method and proposed 51 features of HTs, including logic-gate fanins, flip-flops, multiplexers, loops in a netlist, constants, and levels to primary input and output.

Kurihara et al. [71] proposed 25 HT features based on trigger circuit structure for pre-silicon detection. To improve the RF classifier for large gate-level netlists, the “fan_in_uxdy” feature set was introduced. This feature involves moving down *x* gates to the output side and up *y* gates to the input side, with “fan_in” indicating the number of inputs and “uxdy” representing the path pattern. The fan_in_uxdy contains 25 features, given the trigger circuit’s maximum of 5 levels (1≤x,y≤5).

Xiang et al. [73] applied an RF-based method for side-channel detection, finding that HTs cause detectable fluctuations in nearby power supply voltage, which can be sensed by an RO. The authors collected 500 data points per RO, filtered to 100, and used an RF classifier to analyze the RO output frequencies, achieving 56.7% accuracy.

Existing RF-based HT defense strategies offer distinct advantages in different scenarios. Pan et al. enhanced adaptability by combining static and dynamic features, while Hasegawa et al.’s [70] 51-dimensional structural features suit large netlists but lack targeted optimization. Kurihara et al. [71] focused on trigger circuit structures, improving recognition in specific HT scenarios, but their scalability is limited. Xiang et al. [73] showcased RF’s potential in post-silicon detection through side-channel analysis. Despite their effectiveness, these methods are constrained by feature design and application-specific limitations.

### 6.3. PCA-Based

PCA is a common dimensionality reduction technique that maps high-dimensional data to a lower-dimensional space while preserving the original information. In IC security, data is often high-dimensional and noisy, affecting detection efficiency and accuracy. Many researchers use PCA to reduce feature dimensions and remove noise.

Dong et al. [74] used XGBoost to extract 16 features from 56 potential ones, then applied PCA to reduce the feature space to 2 dimensions. The authors combined the 2D data with the LOF algorithm for unsupervised detection. This approach achieved an AUC of 0.88, compared to 0.85 with factor analysis (FA). The final detection results with PCA and LOF showed a TPR of 73.08% and a TNR of 97.52%.

Huang et al. [75] applied PCA to reduce the dimensionality of FPGA power-frequency data from 23 to 10 using simulated power samples without HTs. To address process deviation, ±5% fluctuations were simulated, generating additional test samples. PCA alone could not completely distinguish logical HTs, so the authors combined it with Mahalanobis distance to improve detection accuracy and identify small logical HTs.

Hu et al. [76] and Nowroz et al. [77] used PCA for HT detection based on thermal and power maps. Nowroz et al. captured thermal maps with infrared imaging and derived power maps from them. Both studies applied two-dimensional PCA to distinguish real chips from those with HTs, effectively detecting small HTs with power consumption as low as 0.05–0.2% of total chip power, even with 40% CMOS variations.

Yan et al. [78] applied PCA to reduce the dimensionality of runtime chip temperature data, where thermal information defined 9 PCA variables, each generated from 40 Monte Carlo simulations. The PCA-based approach effectively detected HTs under various noise levels and process variations, with simulations showing that HTs as small as 2 µW could be detected in circuits with power over 500 µW, based on the thermal profile.

PCA-based HT detection methods aim to reduce data dimensionality, suppress noise, and enhance the separability between normal and malicious patterns. Dong et al. [74] and Huang et al. [75] both apply PCA at the signal level. Dong et al. focus on feature extraction from circuit behavior, while Huang et al. utilize simulated power-frequency data, highlighting the role of PCA in simplifying complex feature spaces for HT detection. In contrast, Hu et al. [76], Nowroz et al. [77], and Yan et al. [78] utilize PCA on post-silicon data such as power and thermal maps, emphasizing spatial anomaly detection under runtime and process variation conditions.

### 6.4. KNN-Based

KNN is an ML algorithm for classification and regression tasks. As an instance-based learning method, it makes predictions using known data samples without model training. Some researchers have used KNN in HT detection.

Mohanraj et al. [80] proposed a side-channel analysis technique for HT detection using power consumption traces, employing a KNN classifier as an evaluator alongside a modified Whale Optimization Algorithm (WOA). Power traces were collected with an oscilloscope, and the WOA was used for feature selection. After testing K values from 2 to 15, K = 5 was selected as the optimal parameter, achieving the highest accuracy and lowest error rate on the test dataset.

In addition, Seum et al. [79] used KNN for HT detection in the pre-silicon stage, identifying potential HTs through software simulation. Lodhi et al. [72] proposed a runtime HT detection based on the power consumption profile of a microcontroller instruction set, implementing the MC8051 microcontroller in VHDL and using KNN to train the model.

Although KNN-based HT detection methods rely on instance-based learning without explicit model training, they differ in detection stages, data types, and application focuses. Mohanraj et al. [80] applied KNN within a side-channel analysis framework, utilizing power traces and feature selection via a modified Whale Optimization Algorithm. In contrast, Seum et al. [79] used KNN in the pre-silicon stage, identifying HTs via simulated behavior. Lodhi et al. [72] focused on runtime detection, using power profiles from a VHDL-implemented MC8051 microcontroller.

### 6.5. K-Means-Based

K-means is a traditional unsupervised learning method that clusters data, grouping similar points together while separating dissimilar ones. In HT defense, some researchers have applied the K-means algorithm to detect and analyze abnormal circuits at both the pre-silicon and post-silicon stages.

Salmani et al. [82] used the K-means algorithm to detect HTs in gate-level netlists. The approach extracted CC0, CC1, and CO features from SCOAP values for each signals. K-means clustered the signals into Genuine Signals, High Controllability and High Observability Trojan Signal. Genuine Signals typically had lower controllability and observability, while Trojan signals exhibited high controllability or observability, designed to affect circuit behavior without easy detection.

In [83], the authors used the K-means algorithm to cluster suspicious signals that may contain HTs in normal circuits. Signals were grouped into five clusters based on imbalance and proximity to rare signals (RareSig). The top cluster had signals likely to be HTs, showing high imbalance and closeness to RareSig, making activation through normal inputs difficult. Conversely, the low cluster contained balanced signals far from RareSig, indicating easy activation through normal inputs.

Bao et al. [84] compared structural area and centroid differences between RE and golden model images to divide the circuit layout into three clusters: Trojan-free (TF), Malicious Addition, and Malicious Deletion. The TF cluster represents layout grids unaffected by HTs, while the Malicious Addition cluster has extra structures in the RE layout, causing area or centroid shifts. The Malicious Deletion cluster shows structural deletions, indicating HT-affected layouts.

Nguyen et al. [85] employed backscattering signals from electromagnetic interactions with circuits to detect HTs. Structural changes, such as Trojan insertion, cause subtle signal variations, which were represented as feature vectors for clustering. This approach groups circuits consistently by their susceptibility to HTs, enabling the distinction between normal and HT circuits.

These K-means-based HT detection methods leverage unsupervised learning to identify anomalies in signals or layouts. Salmani et al. [82,83,85] applied K-means at the signal level, using controllability and observability features, to distinguish between normal and Trojan signals, effectively capturing HTs with high controllability or observability. In contrast, Bao et al. [84] employed K-means at the layout level, detecting structural changes caused by HTs through geometric features such as area and centroid shifts. While differing in focus, all methods demonstrate the strength of K-means in unsupervised HT detection, especially when labeled data is limited.

## 7. Defense Strategies Based on Advanced Machine Learning

HT detection methods based on advanced ML include neural networks, reinforcement learning, ensemble learning, and transfer learning. Neural-network-based defense methods include multi-layer neural networks (MNNs), CNN, artificial neural networks (ANN)s, long short-term memory network (LSTM), GNN, and TextCNN. Table 5 summarizes the HT defense strategies based on advanced ML.

### 7.1. Neural-Networks-Based

Neural networks have played an important role in ML by simulating the brain’s neural systems, driving AI progress in areas like image recognition, natural language processing, and speech recognition. They are widely used in software and hardware, showing great potential in areas like IC security.

#### 7.1.1. MNN-Based

MNN is a multi-layer neural network with an input, one or more intermediate, and an output layer, focusing on the pre-silicon HT detection.

Hasegawa et al. [88] proposed an MNN-based HT detection method for gate-level netlists during IC design. The input layer consists of 11 HT feature, and the output layer has two units for comparison. Experiments showed optimal classification with a middle layer of 200, 100, and 50 units across three layers. In a later work, Hasegawa et al. [89] introduced R-HTDetector, a robust HT detection method based on MNN, using adversarial training on a small adversarial sample set. These methods achieved excellent results.

While both methods are based on MNN architectures and demonstrate strong performance, they differ in terms of robustness and training strategies. The earlier method focuses on architectural design and feature selection, whereas R-HTDetector enhances model resilience through adversarial training.

#### 7.1.2. CNN-Based

CNNs, inspired by biological neural networks, are an excellent type of deep learning model widely applied across various fields, including IC security.

Sharma et al. [90] proposed a deep CNN for HT detection in RE, with two convolutional layers, two pooling layers, and four fully connected layers. Additionally, two metrics were introduced to prevent overfitting: accuracy differential metric (ADM) and loss differential metric (LDM).

Yu et al. [91] applied a customized LeNet-5 CNN for HT detection on non-image data using embedded Pin-Cell-Pin (E-PCP) vector input. The model includes two convolutional layers, two subsampling layers, three fully connected layers, and one softmax output layer. Convolutional layers extract features, subsampling layers reduce data size and enhance model robustness, and the fully connected and softmax layers calculate and normalize the HT classification score.

Both approaches leverage CNN architectures but differ significantly in design and application. Sharma et al.’s model is tailored for RE scenarios and incorporates custom regularization metrics (ADM and LDM) to mitigate overfitting. In contrast, Yu et al. adapt the classic LeNet-5 architecture to process structured non-image data via E-PCP vectors, emphasizing efficient feature extraction and robustness.

#### 7.1.3. TextCNN-Based

TextCNN is a CNN model for text classification that extracts features through convolution, reduces dimensions via pooling, and uses a fully connected layer for classification. It can handle diverse circuit information that traditional ML algorithms struggle with and can automatically extract features.

Xu et al. [92] proposed HTtext for pre-silicon HT detection using TextCNN. It converts IC netlists into simple path sentences, analyzes them with convolutional, pooling and fully connected layers, and pre-trains word embeddings with Word2vec. HTtext achieved an average accuracy of 99.26% in experiments, demonstrating efficiency and stability with the Stability Efficiency Index (SEI).

Chen et al. [93] proposed MHTtext, a TextCNN-based framework for efficient HT detection. It balances accuracy and efficiency through a global strategy that generates comprehensive path sentences from IC netlist and a local strategy that quickly extracts key features. TextCNN processes these strategies using one-dimensional convolutional layers, pooling, and fully connected layers to identify potential Trojans.

Both HTtext and MHTtext utilize TextCNN as the core architecture for HT detection from netlist-derived path sentences, but they differ in strategy and focus. HTtext emphasizes semantic representation through Word2vec pre-training and achieves high detection accuracy, demonstrating the potential of natural language processing in hardware security. In contrast, MHTtext introduces a dual-strategy approach—global for coverage and local for efficiency, making it more adaptable to varying detection requirements.

#### 7.1.4. ANN-Based

ANN is a feedforward neural network without cyclic connections, offering low calculation complexity and cost. As IC scales continuously increase and market competition intensifies, the demand for time-efficient HT detection has led researchers to focus on ANN methods.

Wang et al. [94] proposed DetectANN, an ANN-based HT detection for NoC. Using data generated by the Gem5 simulator, which included a runtime error injection module to simulate transient errors, the authors extracted 12 attributes related to these errors, such as buffer and link utilization, and local temperature, etc. These attributes served as input for an ANN with a single hidden layer of 30 neurons, trained for binary classification to identify HT-infected NoC nodes.

Compared to CNN or TextCNN-based methods, which focus on structural and semantic feature extraction from netlists, DetectANN leverages runtime behavioral features in NoC environments, highlighting ANN’s strength in low-complexity, real-time analysis. While its simplicity ensures computational efficiency, the model’s reliance on simulator-generated transient error data may limit its generalizability to other IC contexts or HT types. Moreover, the use of shallow architectures could restrict its ability to capture complex patterns compared to deeper neural models, such as CNNs or MNNs.

#### 7.1.5. ELM-Based

Wang et al. [95] applied ELM for side channel analysis in HT detection, using an FPGA board and Tektronix DPO7104C oscilloscope and P6139A for data collection. The experiment evaluated three data groups with different implanted Trojans (0.81%, 0.40%, and 0.15%) and achieved average detection success rate of 100%, 96.899%, and 89.549% after training.

As a specialized form of ANN, ELM offers faster training due to its randomized hidden layer weights and analytical solution for output weights. Compared to DetectANN introduced in the previous section, which relies on runtime behavioral features, ELM focuses on side-channel information, leveraging physical signals for HT detection. This makes it particularly suitable for post-silicon analysis. However, the model’s dependency on high-quality side-channel data and specialized measurement equipment (e.g., oscilloscopes) may limit its practicality in large-scale or resource-constrained scenarios.

#### 7.1.6. MLP-Based

MLP is also a type of ANN with an input layer, multiple hidden layers, and an output layer.

In [96], MLP is used to detect abnormal power consumption in an SiP chip, integrated into an FPGA. Power noise traces from the SiP were collected using a time-to-digital converter. The network structure included a 16-neuron input layer, two hidden layers with 64 and 32 neurons, and a single-neuron output layer for HT identification. The trained model’s floating point parameters were quantized into fixed-point data types for efficient FPGA implementation.

In [97], MLP is used to detect the power impact of untrusted IP on trusted IP, which can reveal the HTs. The authors used multiple current mirrors to perform fine-grained power profiling, and a single power port acquisition block to measure current for each instruction pipeline stage. Current data was converted to power data, with instructions and categories as input features. The network comprised a three-neuron input layer (power, instruction, category), two hidden layers, and a single-neuron output layer to detect HTs in the untrusted IP.

MLP demonstrates versatility in HT detection across various hardware platforms and feature modalities, from power noise traces in SiP chips [96] to fine-grained power profiling of IP cores [97]. Its relatively simple architecture enables efficient implementation on FPGA through parameter quantization, balancing detection accuracy and hardware resource constraints. However, due to its feedforward nature and lack of inherent spatial or temporal feature extraction capabilities, MLPs may require careful feature engineering to maintain robustness against diverse and evolving HT attack vectors.

#### 7.1.7. LSTM-Based

LSTM, a type of RNN with recurrent connections, is well suited for processing sequence data like text and time series. In IC design, RTL-level and gate-level netlists are treated as sequence data, enabling LSTM to be effectively applied to pre-silicon HT detection. Unlike traditional RNNs, LSTM overcomes gradient vanishing and exploding, enabling it to learn long-term dependencies in HTs, which may embed complex logic activated after a long delay.

Lu et al. [98] used a stacked LSTM network to detect Trojans from netlist data by converting the netlist into a directed graph with gates as vertices and connections as edges. The n-gram model formed gate-level n-gram sequences, while an Order-Sensitive Co-occurrence Matrix captured relationship between gates and generate vector representations. The LSTM processed these gate-level sequences through three stacked layers to capture temporal dependencies, with the output layer predicting the likelihood of each gate sequence being HT.

Yu et al. [91] applied LSTM to pin-level netlist data, converting it into a directed graph with the “Cell-Pin Splitter” module. The breadth-first search (BFS) algorithm then extracts local netlist blocks, providing structured information. A skip-gram model generated an E-PCP with vector representations of PCP sequences. The LSTM, with two layers and a 128-dimensional hidden state, processes the E-PCP vectors and uses the final hidden state for sequence representation. The network included a fully connected layer and a softmax output layer to calculate the classification score and normalize it to the probability of HT.

LSTM can be combined with other ML models for HT detection. In the zero-shot learning approach by Pan et al. [99], LSTM acts as a memory, working with a graph convolutional neural network (GCN) to store general knowledge of HTs. During the reasoning stage, LSTM uses this general knowledge to evaluate new hardware designs and, together with the similarity score between the circuit and HT, aids in making the final classification decision.

These works demonstrate the flexibility of LSTM in handling netlist data at different abstraction levels—from gate-level n-gram sequences to pin-level vector representations—and in combination with graph-based models for enhanced reasoning. While Lu et al. [98] and Yu et al. [91] focus on structural sequence modeling, Pan et al. [99] extend LSTM’s role to knowledge retention in a zero-shot learning framework. Despite their differences, all methods leverage LSTM’s strength in capturing long-range dependencies, making it a promising tool for detecting stealthy HTs with delayed or complex triggers.

#### 7.1.8. GNN-Based

In the IC design stage, RTL-level and gate-level netlists describe circuit connections as irregular text data representing graph structures. In the previous sections, most ML methods in HT defense solution can only find robust feature representations for non-Euclidean data in text form. However, traditional ML methods are good at processing Euclidean data with regular structural characteristics. Therefore, to better handle the irregular netlist data in the IC, a GNN can be used to model and train the irregular circuit topology.

In the GNN4IP model, Yasaei et al. [100] used a graph neural model to solve the problem of IP core piracy detection. They converted the circuit topology connection relationship inside the IP core into a data flow graph, used a GCN to generate a graph embedding vector, and finally compared the similarity between two IP cores to determine whether there is IP core piracy.

In the GNN4TJ model, Yasaei et al. [101] used the same method as the GNN4IP model to generate a data flow graph DFG in the RTL-level netlist that describes the circuit topology connection relationship and used a graph convolutional network to generate graph embedding vectors to achieve circuit feature extraction. Finally, a multi-layer perceptron was used for classification to achieve HT detection.

Cheng et al. [102] used the GNN4Gate model for HT detection. In this model, a bidirectional GNN was used to extract circuit features and aggregate the information of neighbor nodes from both the propagation and dispersion directions of circuit signals, enabling the model to more comprehensively understand the structure and behavior of the circuit.

Cheng et al. [103] proposed the GateDet model, which considers that circuit diagram information can only be aggregated in one direction during feature extraction using the GNN model, and the perception range of the node is limited. Therefore, the authors introduced a timely information fusion strategy to maintain the high accuracy of HTs and improve the generalization ability of the detection model.

Chen et al. [104] proposed the GNN4HT model, utilizing a two-stage strategy for multi-classification of HTs. In the first stage, a graph isomorphic network was used to locate the HT and extract the HT subgraph information. In the second stage, the HT subgraph information was trained by the GNN model, and, finally, the multi-classification of HTs was achieved. Based on the above examples, it is not difficult to find that the GNN can be regarded as a feature extractor, but its processing object is graph structure data. The features extracted by it can be used to achieve node classification, graph classification, etc.

These GNN-based methods have been widely applied, but they differ in focus and design. GNN4IP and GNN4TJ operate on RTL-level data flow graphs for IP piracy and HT detection, while GNN4Gate and GateDet improve feature aggregation through bidirectional and timely fusion strategies. GNN4HT further advances the field with a two-stage framework for multi-class HT classification. Collectively, they show that GNNs excel at capturing the structural dependencies in circuit topologies, but challenges remain in handling large-scale designs and integrating functional or temporal information.

### 7.2. Reinforcement-Learning-Based

Reinforcement learning has made significant progress in optimizing decision making through trial-and-error and reward mechanisms, enabling intelligent systems to learn and adapt autonomously. With the evolution of deep reinforcement learning, it shows great potential across various fields, including HT detection, where it enhances accuracy and efficiency by optimizing logical test vectors.

Pan et al. [105] proposed TGRL, an HT detection method that uses reinforcement learning to generate test patterns by combining rare signal stimulation with SCOAP values. The environment is a circuit netlist, with the state defined by rare signal and SCOAP values. Actions involve flipping bits in test vectors randomly, and rewards are based on rare signals and the SCOAP reward. The process includes initialization, parameter setting, inner loops and updates. Through iterative training, TGRL efficiently generates test vectors, improving trigger coverage and reducing generation time.

Chen et al. [106] proposed AdaTest, a detection method combining reinforcement learning and adaptive sampling to generate logic test vectors. Unlike [105], AdaTest defines the state as the current test set and the action as selecting new test vectors to improve HT detection. The environment, a circuit netlist, provides rewards based on DAG-level diversity, rare node activation, and SCOAP testability. AdaTest generates candidate test inputs, evaluates them with an SAT solver, and updates the test set with high-reward inputs until effective HT detection is achieved.

Both TGRL and AdaTest leverage reinforcement learning to optimize logic test vector generation, but differ in how they define states, actions, and reward mechanisms. TGRL focuses on bit-level flipping guided by rare signal activation and SCOAP, emphasizing fine-grained exploration. In contrast, AdaTest adopts a more strategic, set-level sampling approach, using DAG-level diversity and SAT-based evaluation to refine its test set.

### 7.3. Ensemble-Learning-Based

Ensemble learning improves performance by combining multiple models, with XGBoost being a representative. XGBoost provides tools for feature importance evaluation and balances HT dataset imbalances through weight adjustment, making it ideal for pre-silicon HT detection.

Dong et al. [108] proposed a gate-level HT detection method using XGBoost, optimizing recall and F-value metrics to derive 49 effective features from 56 initial ones. The XGBoost classifier uses an objective function that includes both a loss function and a regularization term. Experiments demonstrated an average recall rate of 89.84% and an average F-value of 86.75% with the selected features.

Sharma et al. [107] proposed a class-weighted XGBoost (CW-XGB) method for HT detection using SCOAP values. SCOAP values (CC0, CO, SO, etc.) were extracted from the gate-level netlist, and the best features—CC0, CO, and SO—were selected using permutation importance (PI). The CW-XGB model was trained with 1500 estimators, a max depth of 3, and a minimum child node weight of 1. The model demonstrated superior accuracy, precision, and recall on 16 Trust-Hub benchmarks.

Negishi et al. [69] proposed a gate-level HT detection method using a gradient-boosted decision tree model. They optimized the hyperparameters of XGBoost and constructed an HT detection method based on XGBoost using the optimized hyperparameters. The experimental results showed that the F-value obtained in the XGBoost model was 0.797, which was further improved to 0.842 with tuning.

These XGBoost-based methods all leverage the algorithm’s strengths in handling imbalanced datasets, evaluating feature importance, and providing strong predictive performance. Dong et al. [108] emphasize feature selection and metric optimization, while Sharma et al. [107] enhance the model’s sensitivity to minority classes through class weighting and SCOAP-based features. Negishi et al. [69] focus on performance tuning via hyperparameter optimization. Despite their different emphases, all three approaches demonstrate the effectiveness of gradient-boosted decision trees in pre-silicon HT detection. However, they rely heavily on feature selection, which may limit their adaptability to unknown or obfuscated Trojans.

### 7.4. Transfer-Learning-Based

Transfer learning can reduce training time while maintaining accuracy. By leveraging a pre-trained source domain model, it also alleviates data sparsity issues to a certain extent. With the development of deep learning, transfer learning has been further promoted. The following will introduce some research based on transfer learning in the field of HT defense in recent years.

Faezi et al. [109] adapted a model for runtime HT detection using Golden-free self-reference technology. Two models were created: the reference model, trained on benchmark data, and the target model, trained on target data without the softmax layer. Both models share weights for single-class feature extraction. During training, parameters from the pre-trained model are shared, with the convolution layer frozen and a small learning rate (0.000001) to prevent deviations. The model uses OC-SVM, KNN, and LOF for Trojan detection from bypass signals.

Sun et al. [110] adapted the VGG-16 network from computer vision for time-frequency HT detection in electromagnetics during side-channel analysis. The original one-dimensional electromagnetic signals from probes needed conversion to a two-dimensional format for VGG-16, accomplished using continuous wavelet transform (CWT) to generate RGB images of time-frequency data while filtering noise. The images were resized to 224 × 224. Using a pre-trained VGG-16 model, the authors performed feature extraction, freezing some parameters and modifying the fully connected layer for HT detection. SVM was employed as the final classifier, leveraging its structural risk minimization principle for improved generalization.

Both methods utilize transfer learning to improve HT detection efficiency and accuracy, but focus on different data types and adaptation strategies. Faezi et al. [109] emphasize weight sharing and fine-tuning on runtime bypass signals with classical anomaly detectors, while Sun et al. [110] adapt a vision-based model by transforming side-channel electromagnetic signals into images for feature extraction. Despite their different approaches, both demonstrate the effectiveness of transfer learning in addressing data scarcity and enhancing model robustness.

## 8. Challenges and Future Work

**Figure 4:**
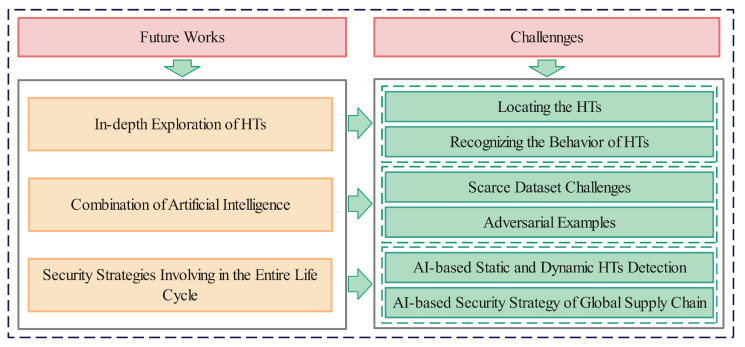
Challenges and future work in IC security.

Besides the issues mentioned above, this section further proposes key trends and challenges for the future, and presents them from three different perspectives, covering in-depth exploration of HTs themselves, the combination of AI technologies, and security strategies involving the entire life cycle of IC. As shown in Figure 4.

### 8.1. In-Depth Exploration of HTs Themselves

The development of AI has driven research to explore the problem of HT future defense strategies, which can be summarized as HT positioning and behavior recognition.

#### 8.1.1. Locating the HTs

HT localization is a critical aspect of the IC security defense system. For netlists flagged as potentially containing malicious circuits, localization provides crucial insights for the subsequent handling of HTs. Currently, HT localization can be categorized into two categories: coarse-grained and fine-grained localization.

Coarse-grained localization typically focuses on the post-silicon stage, leveraging AI techniques to approximate the location of malicious circuits within an IC. These methods combine ML with approaches that analyze internal dynamic signals of the circuit, allowing for an initial localization of the Trojan on a circuit scan image. In one instance of coarse localization, Wen et al. [111] introduced a non-destructive approach using inception neural networks (INNs) to analyze optimized thermal maps collected from normal and Trojan-inserted chips. While such AI-driven methods enable non-destructive localization, they lack structural awareness and often yield vague localization regions, limiting their effectiveness in HT mitigation.

Fine-grained HT localization involves inspecting the internal wires and basic components of a circuit to determine which elements belong to the Trojan. The localization results can pinpoint the exact logical structure of the Trojan and clearly delineate the boundaries between Trojan and normal circuit wires, providing a foundation for further analysis of the Trojan’s functionality. In fine-grained localization, Cheng et al. [103] proposed a detection framework based on Bi-GNNs and directed circuit graph sampling, which identifies sets of gates that are likely to contain HTs, thereby enabling a fine-grained localization of the Trojan.

In summary, vague and coarse-grained HT localization offers little substantive assistance for the subsequent handling of HTs. Additionally, the accuracy, completeness, and precision of such localization cannot be guaranteed, making it challenging to delineate the boundaries between Trojan and normal circuits accurately. AI techniques offer promising solutions by enhancing representational capacity, integrating heterogeneous data sources, and enabling interpretable decision making. These strengths create opportunities for further research, such as developing multi-modal learning frameworks that combine side-channel information (e.g., thermal, EM) with structural circuit data, and designing interpretable models that can explicitly delineate the boundaries of malicious logic.

#### 8.1.2. Recognizing the Behavior of HTs

Recognizing the behavior of HTs plays a pivotal role in detecting various abnormal activities and marks a significant breakthrough in security defense. Traditionally, most HT detection methods are limited to binary, generalized assessments of “yes” or “no”. However, to delve deeper into the core of detection tasks and enable more targeted defenses, pre-silicon recognition of HT malicious behavior presents an effective approach. In existing multi-class detection of HT, the GNN4HT model proposed by Chen et al. [104] employs a GNN to classify extracted Trojan-related information graphs, successfully recognizing HTs that change functionality, denial of service, or leak information.

At present, research on the recognition of malicious behavior in HT is scarce. Moreover, in circuits influenced by multiple types of Trojans, only a single type of HT can typically be detected. Therefore, precise multi-class detection of HT will be a key focus of future work. Meanwhile, AI offers promising solutions due to its strong capabilities in behavior modeling, capturing subtle data patterns, and supporting multi-label classification. Accordingly, future research could leverage AI techniques to systematically summarize and extend existing Trojan types, capture Trojans with overlapping malicious behaviors, and research high-precision HT multi-classification detection models.

### 8.2. Combination of Artificial Intelligence

As AI-based HT defense technologies continue to evolve, challenges like scarce datasets and adversarial samples in models are gradually emerging.

#### 8.2.1. Scarce Dataset

Currently, the field of HT detection faces the challenge of small-scale benchmark training datasets for detection models, leading to overfitting and underfitting issues that negatively impact classification performance. To alleviate the dataset dilemma in HT detection, researchers have proposed various data augmentation techniques, categorized into netlist-level data and method-level data augmentation.

Netlist-level data augmentation enhances RTL-level and gate-level netlist data by generating diverse circuit implementations and test cases. For instance, Hasegawa et al. [112] proposed a logical equivalence method that enhances dataset by equivalently replacing gates within the circuit. At the same time, to replace logic-equivalent circuits within the original circuit, the method also introduces two circuit replacement algorithms, random replacement and partial replacement, to augment the dataset and improve the performance of HT detection.

Method-level data augmentation employs ML methods to implant HTs into portions of the circuit network or generate feature samples similar to HTs after the feature extraction of the detection model is completed. Srinivas et al. [113] use Conditional Tabular Generative Adversarial Networks (CTGANs) and the Synthetic Minority Oversampling Technique (SMOTE) to generate HT features. Tang et al. [114] proposed a dual discriminator-assisted conditional generation adversarial network (D2ACGAN) to generate HT information of the side channel, enabling differentiation of side channel information with or without HTs. Sarihi et al. [115] proposed a reinforcement-learning-based framework that identifies trigger networks and connects them to load modules, enabling automatic HT implantation. This framework employs SCOAP parameters and proximal policy optimization to optimize HT placement, and its generated datasets aid in HT detection training and testing.

#### 8.2.2. Adversarial Examples

Existing HT detection models based on ML methods rarely consider adversarial samples. As a result, the trained models may be affected by adversarial samples. Adversarial samples refer to adding carefully designed small perturbations to the input data. These perturbations do not affect human vision or perception but can cause machine learning models to make incorrect predictions or classifications. Therefore, they are often used to attack and destroy ML models. In the field of HTs, the current method of implanting adversarial examples (AEs) is mainly to make small, carefully designed modifications to the circuit during the design phase so that these modifications have little effect on the performance of the circuit (such as power consumption and delay), but are enough to cause the HT detection model based on ML to misjudge.

Nozawa et al. [116] proposed an adversarial sample generation framework for HT detection. The framework replaces HT circuits with logically equivalent circuits, making them difficult to detect. On this basis, Hasegawa et al. [89] proposed an HT detection method based on adversarial training. By using MNN to train a training set containing adversarial samples, an HT detection model that can detect adversarial samples was implemented.

To solve the adversarial sample problem in HT detection, future work will focus on developing more robust detection techniques and using adversarial training to improve the detection ability of the model.

### 8.3. Security Strategies Involving in the Entire Life Cycle

Throughout the IC life cycle, AI-based static-dynamic HT detections, along with AI-based security strategies for the global supply chain, will be key areas of future exploration.

#### 8.3.1. AI-Based Static-Dynamic HTs Detection

In HT detection, the combination of static and dynamic analysis provides insights from different perspectives, compensating for the limitations of relying on a single approach.

Static detection primarily focuses on the RTL netlist and gate-level netlist during the pre-silicon stage, as well as RE during the post-silicon stage. AI-based static detection identifies hidden malicious circuits by analyzing the static structure of the design, without the need for a golden chip reference. However, static detection struggles to detect HTs that exploit structural changes during circuit operation, as it lacks the runtime data that dynamic analysis provides.

Dynamic detection, on the other hand, is centered around pre-silicon circuit simulation and post-silicon runtime analysis, often through side-channel analysis. By collecting real-time information during circuit operation or simulation—such as power consumption, current, and voltage—it can identify hidden HTs. Nevertheless, current dynamic detection methods typically require a golden chip for comparison. Given the difficulty in obtaining a standardized golden chip, the accuracy of dynamic detection faces significant challenges.

During circuit operation, the propagation of signal values is governed by the logic gates of the nodes. A low signal switching activity often indicates the dynamic features of HTs, as HT triggers typically require rare activation conditions. By combining AI-based static and dynamic HT feature detection, it is possible to perform multi-dimensional analysis of HTs without relying on a golden reference. This approach enables a more comprehensive assessment of potential threats within integrated circuits by integrating insights from static circuit structures with runtime data from dynamic analysis. Therefore, a potential research direction is how to fuse multi-view dynamic and static HT information effectively.

#### 8.3.2. AI-Based Security Strategy of Global Supply Chain

As ICs continue to scale up to tens of billions and beyond, global supply chains and third-party tools, such as 3PIP cores and EDA tools, have become essential in IC design and manufacturing. However, this model creates favorable conditions for attackers to implant malicious circuits within ICs. To mitigate this risk, security strategies for the entire life cycle have become increasingly important.

In existing security strategies, Dong et al. [21] proposed using diversity and randomness strategies for 3PIP to achieve secure production. The IP diversity strategy suggests incorporating IP cores from different suppliers, and the IP randomness strategy advocates for randomly selecting 3PIP during the design phase to prevent collusion attacks among suppliers. However, these security strategies overlook compatibility issues among IP cores provided by different suppliers, and the random selection of IP cores may increase uncertainty in the supply chain, making it difficult to effectively trace the suppliers of malicious circuits in the design.

Currently, existing IC security strategies have limitations and lack entire life cycle control and management. With the continuous development of AI in the IC field, there are substantial benefits for IC defense. Therefore, exploring AI-based security strategy of global supply chain is highly constructive, and integrating technologies such as federated learning, blockchain, and secure multiparty computation can make it feasible to achieve trustworthy, traceable, and accountable IC design and manufacturing.

## 9. Conclusions

Human life is increasingly immersed in CPS, and the low-cost and high-efficiency-driven global supply chain design and manufacturing model highlights the critical importance of IC security. Considering the issues of ultra-large-scale circuits, traditional methods have become ineffective in terms of computational complexity. AI is a feasible and effective way to implement IC security strategies. For the first time, the current status of the combination of IC security and AI technologies is discussed here. This paper aims to make more researchers pay attention to the issues of IC security and attach importance to AI, and its initial and successful combination with the issues of IC security, hoping to promote further in-depth research on related issues.

## Figures and Tables

**Table 1:** Summary of common terms and abbreviations.

Term	Abbreviation
Integrated Circuit	IC
Hardware Trojan	HT
Reverse Engineering	RE
Artificial Intelligence	AI
Machine Learning	ML
Support Vector Machine	SVM
Random Forest	RF
Principal Component Analysis	PCA
K-Nearest Neighbors	KNN
Multi-layer Neural Network	MNN
Convolutional Neural Network	CNN
Artificial Neural Network	ANN
Long Short-term Memory Network	LSTM
Graph Neural Network	GNN

**Table 2:** Integrated circuit scale classification.

Scale Name	Abbreviation	Number of Transistor
Small-scale Integrated Circuit	SSI	No more than 10
Medium-scale Integrated Circuit	MSI	[10,1000]
Large-scale Integrated Circuit	LSI	(1000,10000]
Very-large-scale Integrated Circuit	VLSI	(10000,1000000]
Ultra-large-scale Integrated Circuit	ULSI	(1000000,10000000]
Giga-scale Integrated Circuit	GSI	more than 100 million

**Table 3:** A Summary of the Major HT from Trust-Hub [53,54].

Type	HT	Implanting Phase	Activation Method	Description
Leak Information	AES-T100	pre-silicon	create a code sequence	The AES-T100 Trojan leaks the encryption key via a CDMA covert channel, using PRNG to generate CDMA code, modulating the key bit XOR, and simulating a large capacitor circuit to leak information through the power channel.
	PIC16F84-T300	pre-silicon	specific instruction	The PIC16F84-T300 Trojan is implanted during design and activated by executing a specific number of instructions. When triggered, it manipulates the EEPROM data line to leak secret information in the register.
	S38584-T300	pre-silicon	time interval	The S38584-T300 Trojan is implanted during the design phase and activated at time intervals, combining information leakage with function changes triggered by a counter that detects internal signal conversions when the threshold is exceeded.
Denial of Service	AES-T1800	pre-silicon	predefined input plaintext	The goal of the AES-T1800 Trojan is to accelerate battery drain and shorten battery life. The Trojan is activated after detecting a predefined plaintext and increases power consumption through shift registers, shortening battery life.
	B15-T100	pre-silicon	change the clock frequency	The B15-T100 Trojan causes denial of service by reducing the clock frequency when the 8th to 15th bits of the address line are 0xFF. After activation, the clock frequency is halved, slowing down the circuit operation.
	BASICRSA-T200	pre-silicon	“inExp” signal	The BASICRSA-T200 Trojan disables sender encoding and receiver decoding, causing the relevant modules to fail and achieving a denial of service attack.
	WB_CONMAX-T100	pre-silicon	comparator	The WB_CONMAX-T100 Trojan invalidates subsequent modules by fixing valid bits. It triggers via a comparator with a very low probability ($9.78\times 10^{-68}$). After activation, the highest four bits of the host address bus are fixed to “1”.
Degraded Performance	ETHERNETM AC10GE-T100	post-silicon	always on	The ETHERNETMAC10GE-T100 Trojan is implanted during the manufacturing phase and is always active without any triggering conditions. Its load circuit cause critical path performance degradation.
	MEMCTRL-T100	pre-silicon	combine with software	The MEMCTRL-T100 Trojan combines hardware and software vulnerabilities. The goal is to make the Flash Sleep control bit of the memory controller “1”, so that the flash memory device enters sleep or power-down mode, reducing performance.
	MULTPYRA MID-T100	post-silicon	always on	The MULTPYRAMID-T100 Trojan is implanted during the manufacturing phase, always active, and affects the IC’s critical path by narrowing the network n196.
	S35932-T300	pre-silicon	internal conditional	The S35932-T300 Trojan is implanted during the design phase and activates only in functional mode. It uses a RO load that the slows down the path when oscillating, resulting in performance degradation and denial of service.
Change Functionality	AES-T2300	pre-silicon	special rare signals	The AES-T2300 Trojan is implanted in the AES-128 encryption module. It triggers when s2[89] and s5[121] are both high. After activation, the least significant bit of the encryption output is flipped, making the encryption module invalid.
	B19-T100	pre-silicon	specific vector counter	The B19-T100 Trojan is a combined function-changing HT that includes a trigger and load module. The trigger is a specific vector counter activated within the 100–110 range. The load is an OR gate that can re-integrate the circuit design.
	S15850-T100	pre-silicon	reverse test enable signal	The S15850-T100 Trojan includes two comparators and flip-flops. The comparator drives the clock for the first flip-flop, which feeds into the second. The second flip-flop activates in functional mode by the reverse test enable signal. The load is the MUX of the output port, which leaks internal signals when activated.

**Table 4:** HT Defense methods based on traditional machine learning.

Method	Stage	Description
SVM	pre-silicon	Hasegawa et al. proposed five feature dimensions for pre-silicon stage training, set the features into specific feature vectors and put them into SVM classifier for training. [64]
	post-silcon	Vashistha et al. applied SVM to the physical inspection process. [65]
		Hu et al. applied SVM to side-channel analysis. [66]
		Kulkarni et al. used SVM for runtime detection of multi-core platforms. [67]
RF	pre-silicon	Pan et al. proposed a gate-level HT detection method that combines Shapley value analysis with decision trees. [68]
		Negishi et al. proposed a gate-level HT detection method based on ensemble learning. Using decision trees as the base learner, they achieved high detection accuracy. [69]
		Hasegawa et al. proposed a gate-level network list HT detection method based on RF and proposed 51 features HT. [70]
		Kurihara et al. applied RF-based methods to gate-level IP core detection and subsequently proposed 25 HT features based on trigger circuit structures in [71].
	post-silicon	Lodhi et al. combined the power profile of the microprocessor instruction set with a decision tree to achieve runtime detection of HTs. [72]
		Xiang et al. applied RF-based methods to side-channel detection of HTs. [73]
PCA	pre-silicon	Dong et al. used the PCA technology to reduce the 16-dimensional feature space data to 2-dimensional space data. [74]
	post-silicon	Huang et al. applied PCA to dimensionality reduction of power-frequency data from FPGAs. [75]
		Hu et al. applied PCA technology to the Trojan detection method based on multi-modal heat map characteristics. [76]
		Nowroz et al. applied PCA technology to the Trojan detection method of multi-modal power characteristics. [77]
		Yan et al. applied PCA technology to reduce the dimension of chip temperature data obtained during runtime. [78]
KNN	pre-silicon	Seum et al. used KNN as a trainer for HT detection in the pre-silicon detection stage. They identified potential HTs through software simulation and analysis, thereby improving the security and reliability of IC. [79]
	post-silicon	Mohanraj et al. proposed a side-channel analysis technique based on power consumption traces, which uses a KNN classifier as an evaluator and combines it with an optimization algorithm for detection. [80]
		Yang et al. proposed a side-channel analysis method, in which the KNN algorithm was used as the classifier. [81]
		Lodhi et al. proposed a method for runtime HT detection based on the power consumption profile of the microcontroller instruction set. The KNN algorithm is used to train the model. [72]
K-means	pre-silicon	Salmani et al. used the K-means algorithm to detect HTs in gate-level netlists, achieving no golden model reference. [82]
		Salmani et al. proposed a progressive N-check (GNJ) technique, combined with the K-means algorithm, to reduce the false positive rate (FPR) of HT detection in gate-level netlists. [83]
		Bao et al. combined the K-means algorithm with RE. [84]
		Nguyen et al. applied the K-means algorithm to HT detection in side-channel analysis. [85]

**Table 5:** HT defense strategies based on advanced machine learning.

Method	Stage	Description
MNN	pre-silicon	Hasegawa et al. proposed an HT detection method based on MNN specifically targeting the gate-level netlist in the IC design stage. [88]
		Hasegawa et al. proposed a robust HT detection method (R-HTDetector) based on adversarial training, which was achieved by using MNN to train a training set containing a small number of adversarial samples. [89]
CNN	pre-silicon	Sharma et al. proposed an HT detection technology based on deep CNN for HT detection in RE. [90]
		Yu et al. used a customized CNN model to automatically extract features and perform classification to detect HT using embedded PCP vectors as input. [91]
TextCNN	pre-silicon	Xu et al. proposed an HT detection method based on TextCNN, which can efficiently identify HT node information in the topology. [92]
		Dong et al. proposed a cost-driven TextCNN HT detection method and introduced two strategies: global strategy and local strategy to balance accuracy and computational consumption. [93]
ANN	pre-silicon	Wang et al. proposed an ANN-based HT detection method (DetectANN) for NoC. [94]
	post-silicon	Wang et al. used a special ANN, the ELM, and applied it to the side-channel analysis task of HT detection. [95]
		Zhang et al. applied a feed-forward ANN, MLP model, to abnormal power consumption detection in SiP chips. [96]
		Khalid et al. used the trained MLP model to detect power anomalies generated between microprocessor instruction executions. [97]
LSTM	pre-silicon	Lu et al. used a stacked LSTM network to detect Trojans by extracting vectors of sequential relationships between gates from netlist data. [98]
		Yu et al. used “Cell-Pin Splitter” to convert the netlist into a directed graph, adopted BFS to extract local blocks, used the skip-gram model to generate E-PCP word vectors, and then processed these vectors through a two-layer LSTM network to finally obtain the HT classification probability. [91]
		Pan et al. achieved zero-shot HT detection by combining LSTM and GCN, where LSTM acts as a memory. [99]
GNN	pre-silicon	Yasaei et al. used GNN to convert IP core topology into data flow graphs, generated embedding vectors through GNN, and compared similarities to detect IP core piracy. [100]
		Yasaei et al. used the GNN4IP model to generate a data flow graph DFG and applied a GNN to extract circuit features, and then detected HTs through multi-layer perceptron classification. [101]
		Cheng et al. used the GNN4Gate model and bidirectional GNN to aggregate information from both the propagation and dispersion directions of circuit signals to comprehensively extract circuit features for HT detection. [102]
		Cheng et al. proposed a timely information fusion strategy to solve the problem of the limited node perception range when GNN unidirectionally aggregates circuit diagram information, thereby improving the accuracy of HT detection and model generalization ability. [103]
		The GNN4HT model proposed by Chen et al. adopts a two-stage strategy: in the first stage, a graph isomorphism network is used to locate and extract subgraph information of HTs. In the second stage, the subgraph information is trained through the GNN model to achieve multi-classification detection of HTs. [104]
Reinforcement Learning	pre-silicon	Pan et al.proposed an automated test generation method TGRL for HT detection using reinforcement learning, which aims to generate test patterns by combining rare signal stimulation and controllability/observability analysis. [105]
		Chen et al.proposed a detection method AdaTest that combines reinforcement learning and adaptive sampling for the generation of logical test vectors. [106]
Ensemble Learning	pre-silicon	Negishi et al. proposed a gate-level HT detection method based on ensemble learning. Using decision trees as the base learner, they achieved high detection accuracy. [69]
		Sharma et al. proposed an HT detection technology based on CW-XGB based on the XGBoost model, which uses the optimal SCOAP feature value set to detect Trojans from the gate-level network list. [107]
		Dong effectively screened and constructed 49 new effective feature sets from 56 original features through the XGBoost scoring mechanism to improve the accuracy and efficiency of gate-level HT detection. [108]
Transfer Learning	post-silicon	Faezi et al. applied the HT detection model to new circuits through model transfer technology, and realized real-time detection without reference chips through self-reference technology, improving the practicality and flexibility of detection. [109]
		Sun et al. innovatively applied the deep learning model VGG-16 in the field of computer vision to side-channel analysis, especially in the time-frequency domain of electromagnetic signals, to improve the detection capability of HT. [110]

## Data Availability

The data presented in this study are available on request from the corresponding author.
